# The Treatment of Hand Injuries

**Published:** 1896-08

**Authors:** William Perrin Nicolson

**Affiliations:** Atlanta, Ga., Professor of Anatomy and Clinical Surgery, Southern Medical College, Atlanta


### THE TREATMENT OF HAND INJURIES.*

By WILLIAM PERRIN NICOLSON, M.D., ATLANTA, GA.,
Professor of Anatomy and Clinical Surgery,
Southern Medical College, Atlanta.

In the entire field of surgery there is no class of injuries that more seriously affects the future prospects of the individual than those of the hand, and the subsequent effect upon his capacity for taking care of himself lies largely at the door of the surgeon to whom the treatment is confided. In almost any line of life the earning capacity is impaired by a loss of even a portion of the hand; but, in a large majority of persons, this loss is in direct proportion to the amount of destruction of the member. Hence it becomes the duty of the surgeon not to consider the cosmetic effect, but to strive to leave the earning capacity as little impaired as possible; always remembering that even a small portion, lost or saved, means much in either direction to the patient.

In surgical literature I am sure the special character of the surgery of the hand has not received the attention that the subject deserves, viewed both from its important bearing upon the individual, and the very wide prevalence of such injuries. The teaching of many years ago prevails in many instances. The application of the principles of antiseptic surgery to this class of injuries has enormously increased the field for conservative surgery, and has actually rendered, in my opinion, destructive surgery uncalled for and unjustifiable.

In defining my view of "destructive surgery," as here applied, I would say that when a surgeon goes to any extent beyond the immediate lesion in removing crushed portions of the hand, or in removing any part that is not unequivocally destroyed, he is guilty of destructive surgery. The doctrine formulated by Vernueill some years ago "never to cut away from the hand any tissue that is not pulpified, or destroyed beyond all hope of recovery," has been, in my very large experience, tried hundreds of times, and never found wanting. My faith in the recuperative power of the

*Read by request before the Atlanta Society of Medicine, April 17, 1896.

hand is absolute, when nature is given the opportunity, under suitable surroundings, of reestablishing the injured parts. The well known fact of the enormous blood and nerve supply, and the free communication of the vessels on all sides, has given the hand the power to handle and dispose of offending germs more capably than almost any part of the body; and, at the same time, the excessive amount of fibrous tissue and serous membranes has given to the inflammation, when once begun, the power to extend with alarming rapidity, and to leave excessive stiffening when it has passed away. This great recuperative power, under the proper guidance of the surgeon, will restore parts apparently in a hopeless state of destruction, and will bring forth a useful member from apparent chaos.

From a large observation I do not believe it lies in the power of any man, no matter how experienced, to say what portion of the hand will be restored to life, and what will not, and hence I believe that the decision of the question should be left to the unerring judgment of nature. It may be far more brilliant and beautiful to amputate *secundem artem*, but the patient will poorly appreciate a useless stump, no matter how beautiful. I believe that here we have an instance where the beauty of operating should be ruthlessly sacrificed to the more practical work of conservatism. A physician belonging to the class of a young man who explained to him an unnecessary and improper operation by saying "that he loved surgery too well not to do it," is the most dangerous enemy to the class of patients under observation.

If asked what amount of cutting should be done in a given case, I should say "never use a scalpel in hand injuries." (Vernueill.) Cut away with scissors such tissue as may be hopelessly destroyed, leaving whatever shreds of skin remain to cover stumps, even though skin may slough afterwards. If a finger has been crushed completely off, remove projecting point of bone with bone forceps, covering the end if possible by means of any remaining shreds of skin. The old teaching that surgeon should go far enough beyond the injury to secure a flap of healthy tissue is pernicious, in that it destroys far more of the finger than is necessary. Nature will soon cover such end with granulations, and form skin over it, and should the bone project too much, it can subsequently be removed by a very simple operation. In many instances such a stump may be covered with an organized blood-clot, thus securing an ample bone protection.

Compound fractures should be treated by placing the fragments as nearly in position as possible, and holding them upon splints padded with gauze, or placed in a trough splint and gauze packed around, so as to keep the parts in as good apposition as practical. An excellent substitute for adhesive plaster, and far preferable, is a strip of iodoform gauze, held at each end to the skin by collodion; this will permit the escape of discharges, and admit of applications to the wound, and at the same time secure the parts admirably. I use but few sutures in the hand, and then only at considerable intervals, believing free drainage to be of the utmost importance. Secretions that cannot find ready egress from the wound are backed into tendon grooves, and quickly reach the palm, even ascending the forearm and leaving probably agglutination of the serous surfaces. Irregular gaps are much more safely filled with blood-clots, which can be readily accomplished by the dressings that I shall describe.

The most important requirement in the treatment of such injuries is the securing and maintaining of complete asepsis. It goes without saying that this is a difficult matter, remembering, as we all do, how much painstaking detail is necessary to secure an aseptic condition of the hand of the surgeon. If, in the hands of one whose work naturally presupposes a condition of cosmetic cleanliness, such asepsis is only secured by washings, which, in the language of Dr. Ashhurst, "rivals the washings of the ancient Pharisees," how remote must be the probability of attaining to the same in the hand whose surroundings have been such as to impose upon it conditions which render a vast accumulation of surgical filth necessary and unavoidable. This hand, if prepared for any ordinary aseptic surgical procedure, would require twenty-four hours for perfect cleansing; what must be the prospect then when it is crushed and lacerated, and giving to the unfortunate possessor untold agony? And yet we are told that by using the nail brush with soap and water, soaking the hand in solutions of bichloride of mercury, and by various other devices, we can render such a hand free from germs. Under profound anesthesia this may be possible, but without it I assert that it is not, provided we stop short of brutality. If an aseptic condition is absolutely necessary, and is at the same time so difficult to obtain by the means usually adopted, then if we can secure same by a mode of treatment that is free from danger and absolutely certain in its results, it surely would be a matter of duty to adopt it. These attributes I emphatically claim belong to the continuous saturation of the parts with a harmless antiseptic solution, until beyond the danger from germ invasion.

For the past ten years I have treated practically all hand injuries by the saturation, under rubber tissue, with an antiseptic solution, and to-day my implicit faith in the results is never disappointed. Reasoning would lead to the natural conclusion that any germ life is placed in far greater jeopardy when kept in constant contact with a germicidal solution than when the application has been discontinued, and the remaining spores that may have been left can reproduce and multiply in its absence. Under this treatment I have never seen suppuration occur. Pain is prevented to a large extent by the wet dressings kept warm under the rubber tissue, and circulation is kept from violent reaction. The organization of blood-clot in waste spaces is favored to a high degree; and should any portion, whose life is uncertain, subsequently slough, the separation of the dead portion is hastened, while it is kept aseptic and free from any danger to the patient. The report in the following cases will give an insight into the types represented, and also illustrate the method of dressing which I have followed in these cases:

CASE 1.—*End of thumb entirely severed.*—A. D. A., white, male, ten years of age, while experimenting with a new hatchet, cut off the end of left thumb diagonally through the nail, entirely severing it, and leaving the severed portion in the yard. His mother subsequently searched in the shavings, found the fragment, and replaced it without even washing. When I saw him a short time afterwards the tip was in good position, and adhered, though entirely bloodless. As it had already adhered I did not remove it for the purpose of cleansing the surface, but wrapped around it, along the line of the wound, first a piece of iodoform gauze saturated with camphorated phenol, and over this applied a gauze dressing, covered with rubber tissue. Through an opening in this a solution of listerine in water one to three parts was applied frequently, so as to keep the surface constantly wet. On the fourth day the external dressings were removed, with the exception of the strip which surrounded the line of the wound and was forming a firm support to the part, and similar dressing reapplied. These dressings were continued for one week, when the antiseptic solution was withdrawn, and an ordinary antiseptic dressing applied. The result was that at the end of a few weeks there was perfect union between the parts, not even a line remaining through the nail to show where the separation occurred.

CASE 2.—*End of finger mashed off in a door.*—Mrs. R., while closing folding doors, caught the end of the left index finger, mashing it off just behind the nail, and leaving it attached only by a small strip of tissue. When I saw her, within an hour afterwards, it had already been dressed by another physician, who simply placed around the wound a strip of isinglass plaster. Removing this I found that in placing the parts in apposition there was a rough, ragged gap between the severed portions, which rendered it impossible to bring them in sharp contact. The tip of the finger was placed in as accurate a position as possible, a strip of rubber tissue dipped in camphorated phenol placed along the line of separation; then a strip of iodoform gauze was applied to the skin by collodion on the inside of the finger and passed over the tip to the outer side, where the end was fastened about one inch and a half from the end of the finger. This held the fragment in good position. The gauze dressing was applied under rubber tissue, and the patient instructed to keep the parts thoroughly saturated with listerine solution. Upon the fourth day the removal of the dressings showed the line of separation between the two portions filled evenly by a blood-clot. Wet dressings were continued for about a week, when they gave place to dry antiseptic dressings. The result of this case was the organization of the blood-clot with perfect union of the severed parts and subsequent return of sensation to the tip of the finger.

CASE 3.—*Compound fracture and severe crushing of hand.*—J. C. T., railroad employee, was seen February, 1896, with the following injuries: Right thumb compound fracture, permitting the portion of the first phalanx to be twisted entirely out of position, with the palmar surface turned outward and backward, with a ragged, lacerated wound at the seat of fracture. Beginning at the fissure between the index and middle finger, the hand had been bursted by the force of the blow, forming a lacerated wound about an inch and a half long and extending deeply into the palm. The tips of the middle and ring fingers were both mashed off through the nail. He was seen in the cab of a freight-train, where there was no facility for cleansing the hand. The thumb was twisted into position, wrapped with iodoform gauze, and held so by an aluminum splint, and a gauze dressing applied over the splint. The wound in the palm of the hand was covered by a strip of rubber tissue dipped in camphorated phenol, and approximated by wrapping a strip of gauze around the hand, no sutures being used, the tips of the fingers covered with rubber tissue immediately next the raw surface, a gauze dressing applied, and the entire dressing covered with rubber tissue. This was kept saturated as in the other case for four days with the listerine mixture. Upon changing the dressing the wound in the palm of the hand was found filled with a smooth blood-clot; the tips of the fingers were also covered in the same maner, the thumb appearing in such good shape its dressings were not removed. At the end of ten days wet dressings were withdrawn, and dry antiseptic dressings continued. The thumb remaining in good condition, the original dressing was never removed until the end of the third week, when there was complete healing of the flesh wound, and fairly good consolidation of the bony surfaces, with perfect position of the fragments. At no time during the entire treatment was there any suppuration whatever, though no attempt was made to in any way cleanse the hand before applying the dressing, and the result was perfect recovery, without any stiffening of the hand in any respect.

CASE 4.—*Punctured wound of hand.*—C. B., aged 13, while assisting his father in cleaning his rifle, had the ramrod passed through his hand, entering at the fold of the thumb, passing through to the ulnar border of the hand, where it stopped just under the skin. When seen a short time afterwards there was considerable swelling of the hand, from effusion, and much pain. A wet dressing was applied and kept continually wet for one week, with the result that there was no swelling, no pain, and no evidence whatever of any suppuration, though the instrument which caused this puncture was one that furnished every means for securing this end.

CASE 5.—*Severe laceration of all of the fingers.*—William Singleton, colored laborer, was injured in the summer of 1891 by having his right hand drawn into a corrugated crushing machine of a cotton-seed oil mill. He was seen by me soon after the injury, and it was found upon examination that one corrugation had passed with mathematical accuracy the entire length of the dorsal aspect of each finger, crushing away all of the soft tissues and plowing a furrow in the bone itself. The bottom of each wound showed no trace of either extensor tendon or periosteum. The soft tissues of each finger were compressed into a small strip along the palmar surface, and by using ordinary pressure with the fingers it was found that they could not be replaced so as to meet upon the back of the finger, but left a vacancy of almost half an inch between the edges. It should be said that the entire hand, in fact the entire man, was freely covered with the peculiar colored cotton-seed meal, and perfect cleansing was impossible.

With the assistance of Dr. L. B. Grandy the hand was dressed by the following method: While the tissues were compressed as nearly in apposition as possible, a running catgut suture was passed from side to side so as to hold the edges as nearly in contact as practicable, and leave upon the dorsum of each finger a trough, having for each side bruised tissue, and the bottom bare bone. Over this trough, or groove, was placed a strip of rubber tissue dipped in camphorated phenol; each finger being then separated from the others by several thicknesses of iodoform gauze, and the whole hand enveloped in gauze and bound upon a metallic splint. An outside cover of rubber tissue completed the dressing. An opening was cut into this tissue, and the patient instructed at intervals of several hours to saturate the dressing by pouring through this the following mixture: Carbolic acid one dram, listerine two ounces, water six ounces. Instructions were given that this saturation should be kept up constantly, especially when any pain developed in the wound. These dressings were not changed until the fourth day, and where the groove was upon the back of each finger was found completely filled with moist blood-clot, restoring the contour of the organ and entirely filling up all waste spaces. A similar dressing was applied with antiseptic precautions, and this wetting with antiseptic solutions continued for four days more, at which time the second change of dressings took place. Without going further into the details of the history of the case, I would say that dressings of this character were continued for about two weeks, when it was found that the blood-clot had been entirely replaced by tissue, which was covered with healthy granulations, and these went on to the formation of skin without suppuration. The patient was dismissed from treatment at the end of about six weeks, and since that time has been following his regular occupation in the mill, having good power of flexion and extension, with the hand practically as useful as ever.

The above cases illustrate the principle which I have desired to bring out, showing the influence especially of blood-clot closing up waste spaces in injured hands, and also illustrating the fact that suppuration, even when all conditions seem to favor its occurrence, is prevented by the application of the wet antiseptic dressings. Having advanced in the previous part of this paper the idea of permitting parts that are damaged to slough away under antiseptic precautions, instead of removing them by amputation, I desire to report another case which illustrates the attainment of the desired end of this mode of treatment.

CASE 6.—Mary W., colored, occupied in a steam laundry, while attending a mangle had her hand drawn in between two cylinders heated to a temperature of 350 degrees. It became necessary to send for the engineer and to remove several bolts before her hand could be released, and this occupied about twenty minutes. When I saw her the fingers were flexed, the flesh practically burned away, and what remained was literally cooked and hard, until the hand resembled more a bird's claw. The entire back of the hand was denuded almost to the wrist joint, leaving the metacarpal bones bare. Had an amputation been made at that time it would have been impossible to secure enough flap to cover a wrist-joint stump. I concluded to permit the burned portions to slough away; so the hand was dressed antiseptically with dry dressings, as this was before I began the use of wet dressings in these injuries. After several weeks the line of demarcation was formed, and the fingers were cut away with scissors at the metacarpal phalangeal articulations. The entire back of the hand finally became covered with granulations, skin formed over them, and the only portion of the metacarpus which was lost was the immediate articular ends of the bones of the thumb and index finger. Though the entire process required about two months for a complete cure, this patient recovered with a stump free from sensitiveness, and leaving the entire metacarpus, a fragment of the hand that was of material service to her in earning her living. At no time was there any disturbance from the sloughing which was taking place, and it was kept free from odor by the effects of the antiseptics.

In conclusion, I think it has been demonstrated by these cases, which are selected from a very large number, that the claims which I have made are substantiated by the results. I know of no feature which I more desire to impress than the practical freedom from pain which is attained by this method of dressing, knowing how exceedingly painful any wound of the hand is in almost any form of dry dressing.

As to the antiseptics employed I do not know that there is any especial choice, except that where the application to the wound is to be constantly made I believe that those free from toxic effects should be employed. I have used largely in my work listerine, because at the time I began this work it was about the only representative of this class of antiseptics, and it presents an agreeable odor which is not distasteful to the patient or those about him. Since that time I have used a number of other preparations with practically the same result. Where I desire to add an anesthetic effect I have been in the habit also of combining carbolic acid with the mixture.

The preparation spoken of as "camphorated phenol" is prepared by mixing equal parts by weight of gum camphor and liquefied carbolic acid crystals, or by placing the two bodies together as solids. The resulting product is an oily liquid, of the combined odors of the two constituents, but having properties entirely different. This can be poured into fresh wounds without pain and without any cauterizing effect, not even coagulating blood. It is a powerful antiseptic and deodorizer, and has decided anesthetic value. I have injected as much as fifteen minims into my own forearm without any marked reaction following. In many conditions I regard this as an invaluable preparation.

In defense of the somewhat radical views that I have advanced I beg to say I believe that a trial of this method will bear out the claims made for it in every detail.

